# Pulmonary embolism in United States emergency departments, 2010–2018

**DOI:** 10.1038/s41598-023-36123-2

**Published:** 2023-06-05

**Authors:** Shu-Hsien Hsu, Chia-Hsin Ko, Eric H. Chou, Jeffrey Herrala, Tsung-Chien Lu, Chih-Hung Wang, Wei-Tien Chang, Chien-Hua Huang, Chu-Lin Tsai

**Affiliations:** 1 Department of Emergency Medicine, National Taiwan University Hospital, 7 Zhongshan S. Rd, Taipei, 100 Taiwan; 2 Department of Emergency Medicine, Baylor Scott and White All Saints Medical Center, Fort Worth, TX USA; 3 Department of Emergency Medicine, Highland Hospital-Alameda Health System, Oakland, CA USA; 4 Department of Emergency Medicine, College of Medicine, National Taiwan University, Taipei, Taiwan

**Keywords:** Cardiology, Diseases, Medical research, Risk factors

## Abstract

Little is known about pulmonary embolism (PE) in the United States emergency department (ED). This study aimed to describe the disease burden (visit rate and hospitalization) of PE in the ED and to investigate factors associated with its burden. Data were obtained from the National Hospital Ambulatory Medical Care Survey (NHAMCS) from 2010 to 2018. Adult ED visits with PE were identified using the International Classification of Diseases codes. Analyses used descriptive statistics and multivariable logistic regression accounting for the NHAMCS’s complex survey design. Over the 9-year study period, there were an estimated 1,500,000 ED visits for PE, and the proportion of PE visits in the entire ED population increased from 0.1% in 2010–2012 to 0.2% in 2017–2018 (P for trend = 0.002). The mean age was 57 years, and 40% were men. Older age, obesity, history of cancer, and history of venous thromboembolism were independently associated with a higher proportion of PE, whereas the Midwest region was associated with a lower proportion of PE. The utilization of chest computed tomography (CT) scan appeared stable, which was performed in approximately 43% of the visits. About 66% of PE visits were hospitalized, and the trend remained stable. Male sex, arrival during the morning shift, and higher triage levels were independently associated with a higher hospitalization rate, whereas the fall and winter months were independently associated with a lower hospitalization rate. Approximately 8.8% of PE patients were discharged with direct-acting oral anticoagulants. The ED visits for PE continued to increase despite the stable trend in CT use, suggesting a combination of prevalent and incident PE cases in the ED. Hospitalization for PE remains common practice. Some patients are disproportionately affected by PE, and certain patient and hospital factors are associated with hospitalization decisions.

## Introduction

Pulmonary embolism (PE) is a significant cause of morbidity and mortality worldwide. The annual incidence rate of PE varies widely^1^, ranging from 0.039 per 1000 people in Hong Kong^2^ to 1.15 per 1000 in the United States (US)^3^. Moreover, the incidence of PE has been continuously rising over the past two decades^4–8^. PE patients with high risk had a mortality rate of 25%^9^, and even among stable PE patients, the mortality rate was 0.7%^10^. With the widespread availability of computed tomography (CT) and well-developed diagnostic strategies, many PE patients are now diagnosed in the emergency department (ED)^11,12^.

However, most of the previous PE studies focused on inpatients^7,13–15^, and therefore, the disease burden and clinical management of PE in the ED remain unclear. This knowledge gap is particularly important because the ED serves as a critical point of care for PE patients, where prompt diagnosis and treatment are essential for favorable outcomes. A previous ED study demonstrated that the proportion of PE among US ED patients was approximately 0.08% from 2001 to 2010^16^. In addition, during this period, there was a significant increase in the diagnosis of PE in the ED, along with increased CT utilization. Moreover, a very high percentage of PE patients (86%) in the ED were admitted^16^. Since this report, there have been no updated data on clinical epidemiology of PE in the ED. A later study from the US Nationwide Inpatient Sample revealed a significant increase in hospitalizations with PE from 1993 until 2012^13^. However, whether the rise in hospitalized PE resulted from increased ED visits for PE and/or increased hospitalizations from the ED remains unclear. Taken together, understanding the disease burden and clinical management of PE in the ED is critical for optimizing ED flow and improving patient outcomes.

To address these knowledge gaps, this study aimed to investigate the disease burden (visit rate and hospitalization) of PE in the ED and investigate factors associated its burden in US EDs using the national survey data between 2010 and 2018.

## Methods

### Study design and setting

The NHAMCS is a cross-sectional, multistage probability sample of visits to non-institutional general and short-stay hospitals, excluding federal, military, and Veterans Administration hospitals, located in the 50 states and the District of Columbia^17^. The NHAMCS is conducted annually by the National Center for Health Statistics (NCHS). It covers geographic primary sampling units, hospitals within primary sampling units, EDs within hospitals, and patients within EDs. The number of EDs sampled is approximately 300–400 per year. Trained ED staff collected clinical information during a randomly assigned four-week period for each of the sampled EDs using a structured Patient Record Form (PRF). Data included patient demographics, reasons for the visit, diagnoses, procedures, medications given at the visit, and basic characteristics of the treating physician and hospital. Quality control was performed using a two-way independent verification procedure with a 10% sample of the records. The non-response rate for most items was < 5%. The coding error rates were < 2%^18^. Because the NHAMCS contains publicly available, de-identified data, the ethics committee named National Taiwan University Hospital Institutional Review Board (NTUH IRB) exempted this study from review. All methods were performed in accordance with the Declaration of Helsinki and the experimental protocol was approved by National Taiwan University Hospital Institutional Review Board (NTUH IRB) with the IRB number 202108019W.

### Study population

NHAMCS data from 2010 to 2018 were used in this analysis. First, we excluded ED visits from non-adults aged less than 18 years. Up to five diagnosis fields in the NHAMCS were coded according to the International Classification of Diseases, Ninth Revision, Clinical Modification (ICD-9-CM), or ICD-10. For the current analysis, we used ICD-9-CM codes 415.1.x or ICD-10 codes I26.xx to define PE. These codes have been validated in administrative data^19^. We identified adult visits in which any of the PE codes were listed in any diagnosis field (all-listed) as ED visits for PE. We also conducted sensitivity analyses using visits in which these PE codes were listed in the primary diagnosis field (first-listed).

### Variables

To preserve consistency across years, race/ethnicity was recoded as non-Hispanic white, non-Hispanic black, Hispanic, and other. Insurance was recoded as private, Medicare, Medicaid or other state-based programs, self-pay, and other. The US regions represented standardized geographical divisions, as defined by the US Census Bureau (Northeast, Midwest, South, and West)^4^. Up to five reasons for each ED visit were coded using the Reason for Visit Classification for Ambulatory Care, a standardized sourcebook used in NCHS studies^20^. Chronic comorbid conditions were ascertained based on the PRF, including, but not limited to, diabetes mellitus, hypertension, obesity, cancer, and venous thromboembolism. Data on disease severity and urgency included triage levels, vital signs at triage, and pain scores. We also calculated the simplified Pulmonary Embolism Severity Index (sPESI) to assess PE severity. A number of procedures were documented on the PRF, including endotracheal intubation and cardiopulmonary resuscitation (CPR). Imaging performed in the ED, including computed tomography (CT) scan and ultrasound, as well as laboratory examinations performed, including D-dimer test, were also recorded. Up to eight medications were recorded during an ED visit. The therapeutic category of medication was based on the Multum Lexicon Drug Database^21^. We identified the following PE medications using the Multum codes: (1) heparin (code 261), (2) coumarins (code 262), (3) thrombin inhibitors (code 283), and (4) factor Xa inhibitors (code 285). Visit disposition was recorded for each ED visit. For ED visits resulting in hospitalization, inpatient mortality, and hospital length of stay (LOS) were recorded.

### Outcome measures

The primary outcome measure was the PE visit rate in the ED. This was calculated as the number of adult ED visits for PE divided by the total number of adult ED visits. The co-primary outcome measure was the admission rate for ED visits for PE.

### Statistical analysis

Stata 16.0 (StataCorp, College Station, TX, USA) was used to adjust the variances for the NHAMCS estimates to account for the complex design of the survey. Standard errors (SEs) were calculated for the NHAMCS estimates. All statistical tests were based on estimates that had at least 30 cases and a less than 30% relative SE (i.e., the SE divided by the estimate expressed as a percentage of the estimate) in the sample data according to the NCHS recommendations. For the trend analysis, we combined two years of data to increase the stability of the estimates.

Descriptive statistics are presented as proportions (with 95% confidence intervals) or means (with SEs). We used the weighted chi-square test to analyze the differences between proportions. Logistic regression models were used to test for significant changes in the primary outcomes (ED visit and admission rates for PE) during the study period, in which the calendar year was a linear independent variable. Multivariable logistic regression analysis was performed to assess independent predictors of the two primary outcomes. Due to the limited number of outcomes, the parsimonious multivariable models included age, sex, race/ethnicity, insurance, season, weekend, time of presentation, geographic region, arrival mode, and triage level. As a sensitivity analysis, we replaced the triage level with sPESI and repeated the multivariable analysis because the two variables were collinear. In addition, we repeated all analyses for PE using the first-listed diagnostic codes (presumably more likely to identify new cases). Odds ratios (ORs) are presented with 95% CI. All P values are two-sided, with P < 0.05 considered statistically significant.

### Ethics approval and consent to participate

Since data were evaluated retrospectively, pseudonymously and were solely obtained for treatment purposes, a requirement of informed consent was waived by the National Taiwan University Hospital Institutional Review Board.

## Results

**Figure 1:**
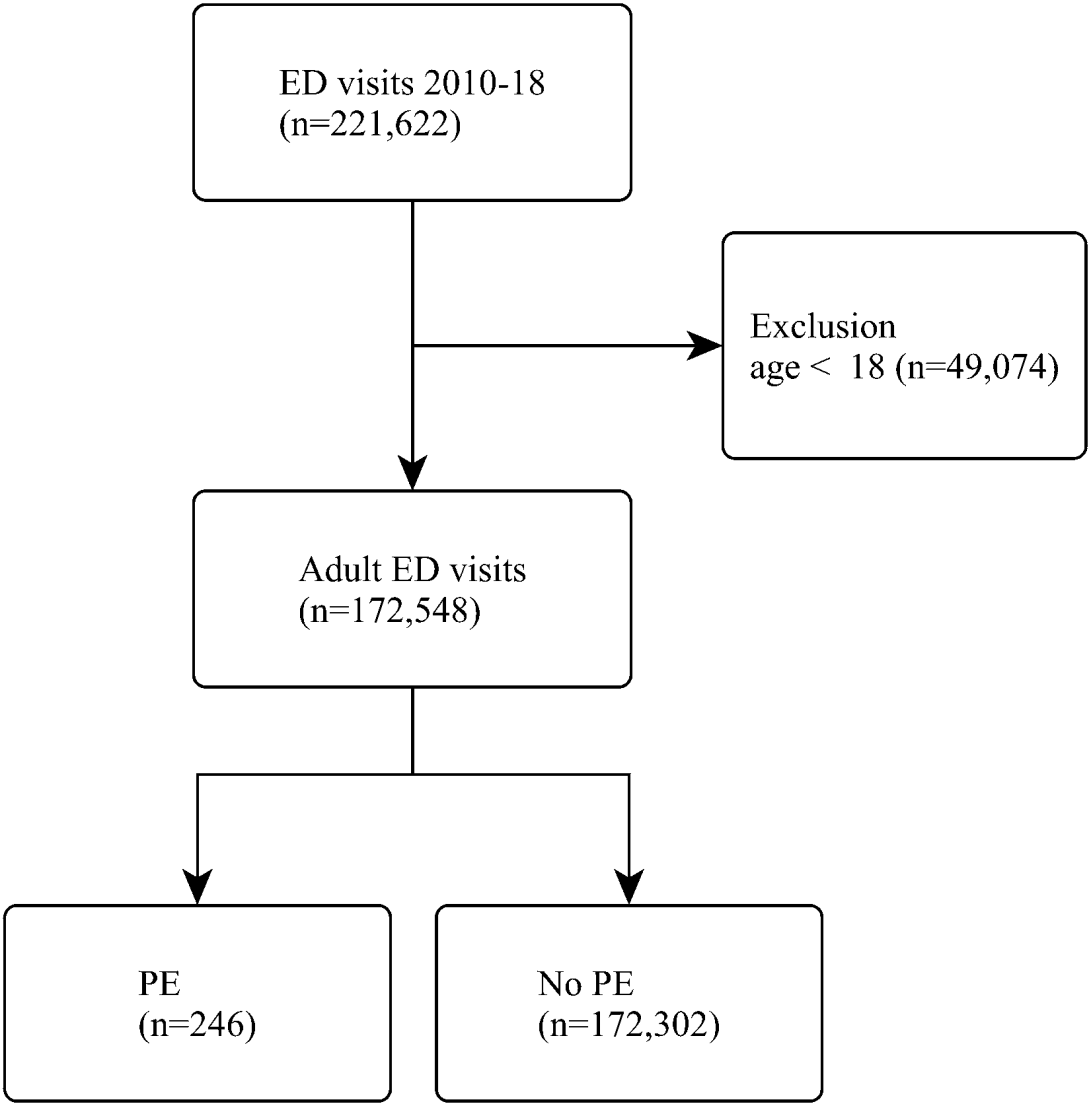
The patient selection process.

A total of 221,622 ED visits were identified from the unweighted NHAMCS database between 2010 and 2018. After excluding patients aged less than 18 years, 172,548 patient visits were included in the analysis. Among these, 246 were ED visits with PE. The patient selection process is shown in Fig. 1.

**Figure 2:**
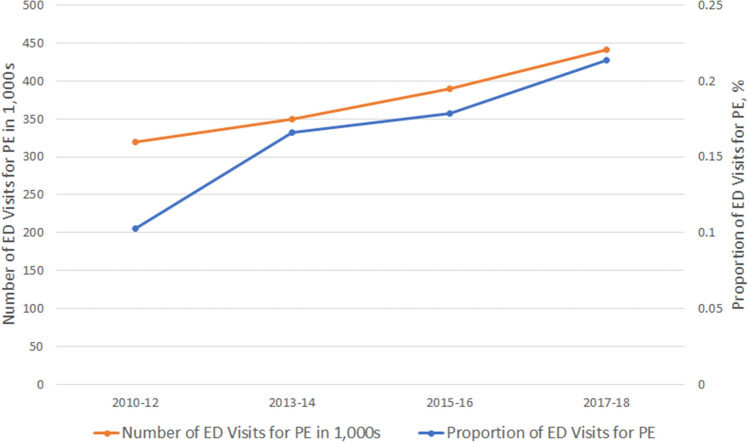
The number and proportion of emergency department visits for pulmonary embolism, 2010–2018.

After weighting, there were an estimated 1,500,000 ED visits for PE over the 9-year study period. Table 1 summarizes the baseline characteristics of these ED visits. The mean age was 56.8 years, and 40.4% were men. The majority of PE patients were non-Hispanic whites (63.8%), followed by non-Hispanic blacks (22.6%) and Hispanics (12.1%). Most of the patients had Medicare insurance (41.9%), followed by private insurance (33.3%), state-based programs (15.2%), and self-pay (7.6%). A larger proportion of PE patients presented to the ED in the fall (35.0%) and during the daytime. Hospitals located in the Midwest had the lowest proportion of the total ED visits for PE (20.7% of all cases), and most of the PE visits occurred in metropolitan EDs (90.7%). Approximately one-third of the patients were sent to the ED by ambulance (34.9%). Regarding the comorbidities among the PE patients, 23.6% had a history of venous thromboembolism (VTE), 14.3% had obesity, 13.7% had cancer, 13.3% had chronic heart failure, and 26.2% had chronic obstructive pulmonary disease. The most common chief complaints were dyspnea (45.5%) and chest pain (25.9%). Most of the PE visits were triaged to level 3 (45.1%), followed by level 2 (40.4%) and level 4 (11.6%). More than one-third of the patients presented to ED without pain (38.0%), whereas 35.0% complained of severe pain. The vital signs at triage showed a mildly elevated heart rate (94 beats/minute) and tachypnea (22 breaths/minute), with lower oxygen saturation (96%). Approximately 57% of the PE patients had an sPESI of one or greater (high-risk). Regarding the individual component of sPESI, 19.6% of the PE patients aged > 80 years, 13.7% had a history of cancer, 27.2% had a chronic cardiopulmonary disease, 20.4% had a heart rate ≥ 110 beats/min, 4.4% had systolic blood pressure < 100 mmHg, and 9.3% had oxygen saturation < 90%. Of patients with PE, 1.2% received intubation, and 0.6% received cardiopulmonary resuscitation. The PE patients underwent a number of examinations: 42.7% received a chest CT scan, 13.9% underwent ultrasound, and 29.3% were tested for D-dimer levels. Regarding medications, most of the patients received heparin (49.8%), 11.2% used warfarin, 8.8% used factor Xa inhibitors, and none used thrombin inhibitors. The mean length of stay in the ED was 6.2 h, Regarding ED disposition, 65.6% of the visits were admitted to the hospital, and 2.5% died in the ED. The mean length of hospital stay was approximately six days. Approximately 28% of the admissions were in the intensive care unit (ICU), and the inpatient mortality was 4.8%. Supplemental Table S1 summarizes the baseline characteristics of ED visits for patients with first-listed PE diagnostic codes. Compared with those in the primary analysis, these patients did not appear to be sicker in terms of comorbid condition or triage level. However, they were more likely to undergo a chest CT scan (66.2%), have a d-dimer test (45.2%), and receive heparin (76.3%) or factor Xa inhibitors (9.1%). Finally, the average admission rate was higher (78.7%) than that in the primary analysis. Figure 2 shows the weighted number and visit rate of ED visits with PE between 2010 and 2018. There were continuously rising trends in both metrics of disease burden. The number of ED visits with PE increased from 319,000 in 2010–2012 to 441,000 in 2017–2018. The mean visit rate was 0.16%, ranging from 0.1 to 0.2%, with an increasing trend (P for trend = 0.002). When repeating the analysis using the first-listed diagnostic codes for PE (47% of the all-listed cases), the increasing trend became statistically non-significant (P for trend = 0.30, Supplemental Fig. S1).

**Table 1:** Baseline clinical characteristics of emergency department patients with pulmonary embolism, 2010–2018.

Variable	Weighted number or weighted mean	Weighted percentage (95% CI)
Overall	1,500,000	
Age group, n (%)		
18–64	882,000	58.8 (49.8–67.2)
65+	619,000	41.2 (32.8–50.2)
Sex, n (%)		
Male	606,000	40.4 (32.7–48.6)
Female	894,000	59.6 (51.4–67.3)
Race/ethnicity, n (%)		
Non-Hispanic white	957,000	63.8 (54.3–72.4)
Non-Hispanic black	339,000	22.6 (15.1–32.4)
Hispanic	182,000	12.1 (7.5–19.0)
Other	22,000	1.5 (0.5–4.4)
Insurance, n (%)		
Private insurance	466,000	33.3 (24.7–43.2)
Medicare	587,000	41.9 (33.1–51.3)
Medicaid or state-based programs	213,000	15.2 (9.8–22.9)
Self-pay (uninsured)	106,000	7.6 (3.8–14.4)
Other	28,000	2.0 (0.9–4.2)
Season, n (%)		
Spring (Mar.–May)	396,000	26.4 (19.0–35.3)
Summer (Jun.–Aug.)	339,000	22.6 (16.8–29.7)
Fall (Sep.–Nov.)	525,000	35.0 (27.2–43.6)
Winter (Dec.–Feb.)	240,000	16.0 (10.6–23.5)
Weekend, n (%)	380,000	25.3 (19.6–32.1)
Time of ED presentation, n (%)		
7:00 am to 2:59 pm	650,000	44.8 (36.0–53.9)
3:00 pm to 10:59 pm	659,000	45.4 (37.0–54.1)
11:00 pm to 6:59 am	142,000	9.8 (5.7–16.4)
Geographic region, n (%)		
Northeast	355,000	23.6 (16.3–33.0)
Midwest	311,000	20.7 (14.3–29.1)
South	450,000	30.0 (21.5–40.0)
West	385,000	25.7 (16.7–37.3)
Metropolitan area, n (%)	1,304,000	90.7 (82.0–95.4)
Arrival by ambulance, n (%)	496,000	34.9 (27.6–43.0)
Number of comorbid conditions, mean (SE)	2.4	2.0–2.8
Comorbidities, n (%)		
Cancer	170,000	13.7 (9.0–20.2)
Venous thromboembolism	266,000	23.6 (15.3–34.6)
Obesity	161,000	14.3 (8.8–22.3)
Chronic heart failure	199,000	13.3 (8.5–20.1)
Chronic obstructive pulmonary disease	326,000	26.2 (17.3–37.6)
Most common chief complaints, n (%)		
Dyspnea	683,000	45.5 (36.9–54.4)
Chest pain	389,000	25.9 (18.8–34.6)
Triage level, n (%)		
1	28,000	2.4 (0.8–7.1)
2	476,000	40.4 (31.5–49.9)
3	532,000	45.1 (35.9–54.6)
4	137,000	11.6 (6.6–19.4)
5	7000	0.6 (0.1–4.3)
Pain score, n (%)		
Severe (7–10)	337,000	35.0 (26.7–44.4)
Moderate (4–6)	208,000	21.6 (14.4–31.2)
Mild (1–3)	51,000	5.3 (2.3–11.9)
No pain (0)	365,000	38.0 (28.7–48.3)
Triage vital signs		
Body temperature, mean, °C	36.7	36.6–36.8
Heart rate, mean, beats per min	94.4	90.1–98.6
Respiratory rate, mean, breaths per min	21.8	19.1–24.6
Oxygen saturation, mean, %	95.9	95.2–96.6
Systolic blood pressure, mean, mmHg	135.1	130.8–139.3
Simplified PESI ≥ 1^a^	79,000	56.8 (47.8–65.3)
Individual component of sPESI		
Age > 80 years	293,000	19.6 (13.3–27.9)
History of cancer	170,000	13.7 (9.0–20.2)
History of chronic cardiopulmonary disease	408,000	27.2 (19.6–36.3)
Heart rate ≥ 110 beats per min	293,000	20.4 (14.2–28.4)
Systolic blood pressure < 100 mmHg	63,000	4.4 (2.2–8.5)
Oxygen saturation < 90%	134,000	9.3 (5.1–16.4)
ED management, n (%)		
Intubation	18,000	1.2 (0.3–4.7)
CPR	9,000	0.6 (0.1–4.0)
Chest CT scan^b^	531,000	42.7 (33.9–52.0)
Ultrasound	208,000	13.9 (9.0–20.9)
D-dimer test	365,000	29.3 (21.0–39.3)
Heparin	747,000	49.8 (40.4–59.2)
Coumarins	168,000	11.2 (6.9–17.6)
Factor Xa inhibitors	133,000	8.8 (4.9–15.4)
Length of ED stay, mean, hours	6.2	5.0–7.4
ED disposition, n (%)		
Admission	983,000	65.6 (55.6–74.3)
Died in the ED	38,000	2.5 (0.6–9.7)
Hospitalization^c^		
ICU admission, %	225,000	27.6 (18.8–38.6)
Length of hospital stay, mean, days	6.3	4.9–7.7
Inpatient mortality, n (%)	44,000	4.8 (2.0–11.1)

*ED* emergency department, *sPESI* simplified Pulmonary Embolism Severity Index, *CPR* cardiopulmonary resuscitation, *CT* computed tomography, *ICU* intensive care unit.

^a^Available in 229 patients.

^b^From 2012 to 2018.

^c^Among those who were hospitalized.

Table 2 shows the factors associated with ED visits with PE. Multivariable analysis confirmed that patients aged 65+ years had a two-fold higher risk of PE ED visits (adjusted odds ratio [aOR] 2.0; 95% CI 1.0–3.8). Obese patients (aOR 2.5; 95% CI 1.3–4.6), patients with cancer (aOR 2.0; 95% CI 1.1–3.7), and patients with venous thromboembolism (aOR 17.4; 95% CI 9.0–33.8) also had a significantly higher risk of ED visits with PE. The Midwest region was independently associated with a 0.5-fold lower risk of PE ED visits (aOR 0.5; 95% CI 0.3–0.9 vs. the Northeast). Supplemental Table S2 describes the factors associated with ED visits with first-listed PE codes. The factors remained similar, except for weekend presentation as a new risk factor and being in the South/West as a protective factor.

**Table 2:** Emergency department visit rates for pulmonary embolism, overall, stratified, and multivariable analysis, 2010–2018.

Variable	Proportion of PE, %	Adjusted OR (95% CI)*
Overall	0.16	
Age group, years		
18–64	0.12	1.0 (reference)
65+	0.32	**2.0 (1.03–3.84)**
Sex		
Male	0.15	0.8 (0.5–1.3)
Female	0.16	1.0 (reference)
Race/ethnicity		
Non-Hispanic white	0.16	1.0 (reference)
Non-Hispanic black	0.16	1.3 (0.7–2.5)
Hispanic	0.15	1.4 (0.6–3.0)
Other	0.08	0.8 (0.2–3.1)
Insurance		
Private insurance	0.18	1.0 (reference)
Medicare	0.27	0.6 (0.3–1.2)
Medicaid or state-based programs	0.10	0.6 (0.3–1.2)
Self-pay (uninsured)	0.09	0.8 (0.3–2.3)
Other	0.06	0.4 (0.1–1.4)
Season		
Spring (Mar.–May)	0.16	1.0 (0.6–1.9)
Summer (Jun.–Aug.)	0.14	1.0 (reference)
Fall (Sep.–Nov.)	0.22	1.5 (0.8–2.7)
Winter (Dec.–Feb.)	0.11	0.8 (0.4–1.7)
Weekend		
Non-weekend	0.16	1.0 (reference)
Weekend	0.15	1.1 (0.7–1.7)
Time of ED presentation		
7:00 am to 2:59 pm	0.16	0.7 (0.4–1.1)
3:00 pm to 10:59 pm	0.17	1.0 (reference)
11:00 pm to 6:59 am	0.10	0.8 (0.4–2.0)
Geographic region		
Northeast	0.22	1.0 (reference)
Midwest	0.14	**0.5 (0.3–0.9)**
South	0.12	0.6 (0.3–1.1)
West	0.19	0.6 (0.3–1.4)
Arrival mode		
Arrival not by ambulance	0.12	1.0 (reference)
Arrival by ambulance	0.29	1.6 (0.96–2.61)
Cancer status		
Cancer	0.58	**2.0 (1.1–3.7)**
No cancer	0.15	1.0 (reference)
History of VTE		
VTE	3.21	**17.4 (9.0–33.8)**
No VTE	0.16	1.0 (reference)
Obesity status		
Obesity	0.60	**2.5 (1.3–4.6)**
No obesity	0.19	1.0 (reference)

*ED* emergency department, *PE* pulmonary embolism, *OR* odds ratio, *VTE* venous thromboembolism.

*Multivariable model adjusts for all variables in the Table.

Significant odds ratios are highlighted in bold.

**Figure 3:**
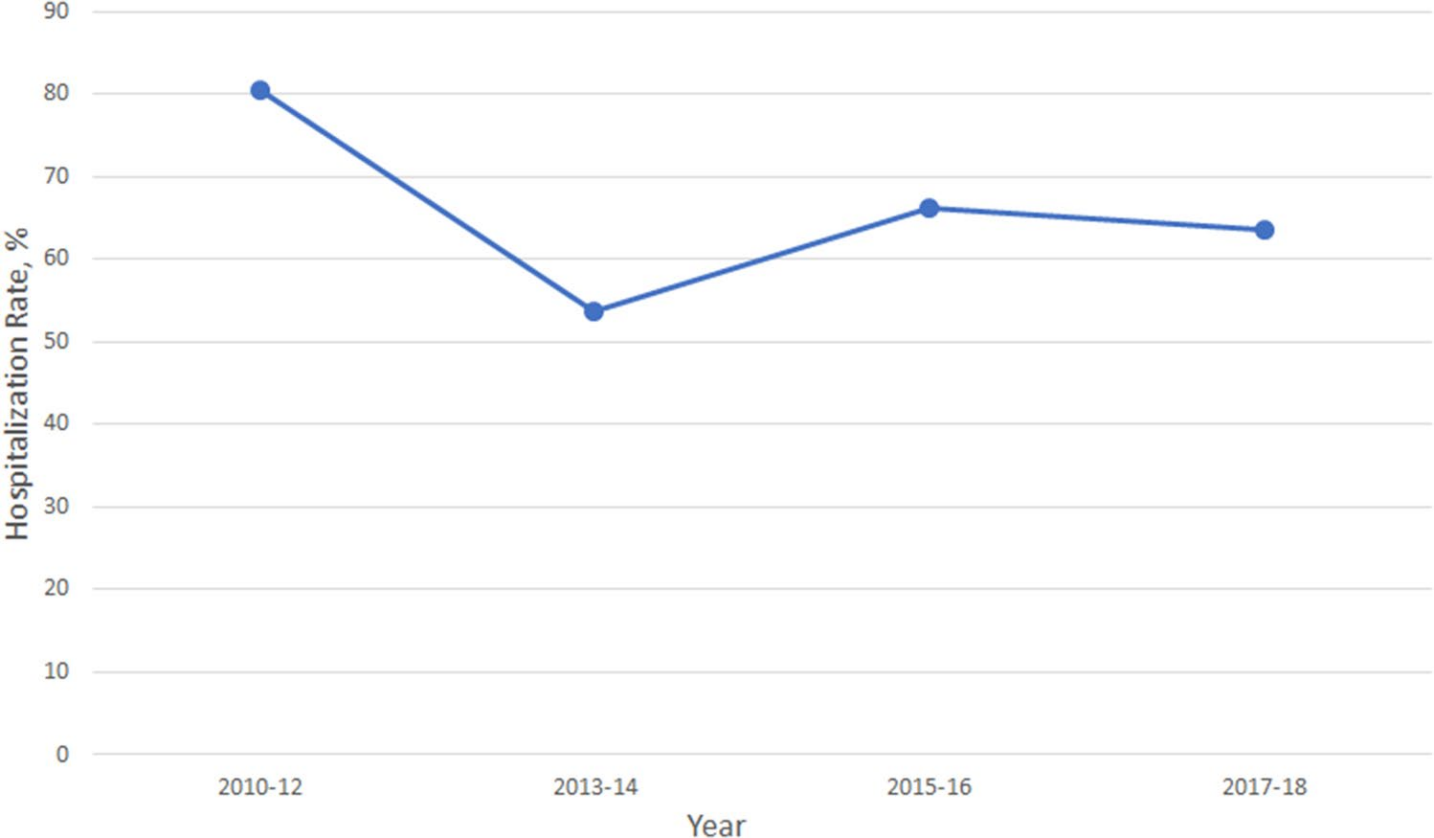
The hospitalization rate among emergency department visits for pulmonary embolism, 2010–2018.

**Figure 4:**
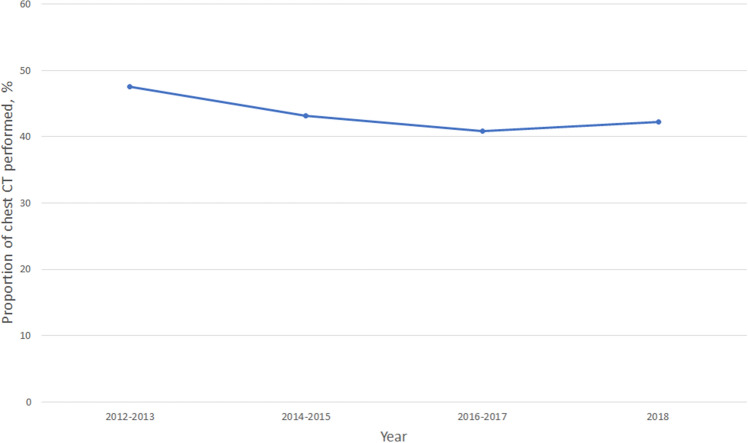
The proportion of chest computed tomography scans performed among emergency department visits for pulmonary embolism, 2010–2018.

Figure 3 shows the admission rate of PE patients in the ED from 2010 to 2018. During the study period, the average admission rate was 65.6%. Admission rates decreased slightly over the study period, but this trend was not statistically significant (P for trend = 0.21). In Supplemental Fig. S2, overall, the admission rate of first-listed PE patients was much higher than that in the primary analysis; however, the admission rate appeared to decrease over time (P for trend = 0.03). Table 3 describes the factors associated with hospitalization among ED patients with PE. The admission rate in men was 77.6% (aOR 4.4; 95% CI 1.9–9.9), representing a 4.4-fold higher risk of admission (vs. women) in multivariable analysis. In multivariable analysis, PE patients presenting to the ED in the fall (aOR 0.2; 95% CI 0.1–0.6) and winter (aOR 0.2; 95% CI 0.1–0.7) were less likely to be admitted than those in the summer. The admission rate during the morning hours was 74.0%, representing a 4.6-fold higher risk of admission in multivariable analysis (aOR 4.6; 95% CI 1.7–12.3 vs. evening hours). Approximately 77.9% of PE patients triaged at levels 1 or 2 were admitted, representing a 3.6-fold higher risk of admission (aOR 3.6; 95% CI 1.2–11.2 vs. triage level 3). By replacing the triage level with sPESI or repeating the analysis using the first-listed diagnostic codes, the multivariable results did not materially change (data not shown). Supplemental Table S3 shows the factors associated with hospitalization among ED patients with first-listed PE codes. The factors identified remained similar to those in the primary analysis. Figure 4 shows the utilization rate of a chest CT scan during the ED visit among patients with PE between 2012 and 2018. The proportion of chest CT performed on patients with PE appeared stable (P for trend = 0.76), ranging from 40 to 47%. The mean utilization rate was approximately 43%. Supplemental Figure S3 shows the utilization rate of a chest CT scan during the ED visit among patients with first-listed PE codes. Overall, the utilization rate appeared higher than that in the primary analysis with a stable trend (P for trend = 0.19).

**Table 3:** Emergency department admission rates for pulmonary embolism, overall, stratified, and multivariable analysis, 2010–2018.

Variable	ED admission rate, %	Adjusted OR (95% CI)*
Overall	65.6	
Age group, years		
18–64	62.2	1.0 (reference)
65+	70.4	1.2 (0.4–3.8)
Sex		
Male	77.6	**4.4 (1.9–9.9)**
Female	57.4	1.0 (reference)
Race/ethnicity		
Non-Hispanic white	64.1	0.6 (0.2–1.6)
Other	66.4	1.0 (reference)
Insurance		
Private insurance	60.4	1.0 (reference)
Medicare	68.3	0.7 (0.2–3.1)
Other	60.4	1.6 (0.5–5.5)
Season		
Spring (Mar.–May)	61.7	0.5 (0.1–1.6)
Summer (Jun.–Aug.)	79.1	1.0 (reference)
Fall (Sep.–Nov.)	65.9	**0.2 (0.1–0.6)**
Winter (Dec.–Feb.)	52.1	**0.2 (0.1–0.7)**
Weekend		
Non-weekend	63.5	1.0 (reference)
Weekend	71.7	1.3 (0.5–3.2)
Time of ED presentation		
7:00 am to 2:59 pm	74.0	**4.6 (1.7–12.3)**
3:00 pm to 10:59 pm	58.2	1.0 (reference)
11:00 pm to 6:59 am	52.5	1.3 (0.3–5.4)
Geographic region		
Northeast	69.6	1.0 (reference)
Midwest	73.6	2.0 (0.4–10.7)
South	65.2	2.0 (0.5–8.7)
West	55.7	1.7 (0.4–7.8)
Arrival mode		
Arrival not by ambulance	65.6	1.0 (reference)
Arrival by ambulance	70.7	0.9 (0.3–2.6)
Triage level		
1 and 2	77.9	**3.6 (1.2–11.2)**
3	63.2	1.0 (reference)
4 and 5	41.0	0.6 (0.2–2.0)

*ED* emergency department, *OR* odds ratio.

*Multivariable model adjusts for all variables in the Table.

Significant odds ratios are highlighted in bold.

## Discussion

By analyzing the US nationally representative data from 2010 to 2018, we identified several important clinical epidemiologic findings. First, the disease burden of PE in the ED continued to increase, despite a stable trend of chest CT use. Second, hospitalization for PE remained common with low usage of direct-acting oral anticoagulants (DOAC). Finally, the decision to hospitalize (or discharge) ED patients with PE involved not only patient but also hospital factors.

Our study indicated a consistently rising trend in PE cases among ED visits. Previous studies revealed that increased PE diagnoses resulted from increased CT adoption over time^13,15,22^. Schissler et al. reported a 58% CT or MRI utilization rate among ED patients with PE between 2001 and 2010, a figure that is higher than our estimate of 43%. The difference might be that our estimate included only CT scans and only the chest part of CT scans. The authors also suggested that the increased imaging usage contributed to the increase in PE cases in the ED^16^. Our study demonstrated a continuously rising trend in PE cases in the ED; however, the utilization rate of CT was unchanged between 2010 and 2018. The continuously rising PE visits in the ED may be a combination of previously diagnosed PE cases and newly diagnosed cases via a CT scan. This was supported by the observation that the increasing trend of PE cases attenuated when we restricted to first-listed (presumably more likely to be new) PE cases. Alternatively, the prevalence of risk factors for PE has increased over time, including aging, cancer, and obesity^23^. Thus, the increase in PE cases in the ED may result from the concomitantly rising trend of these risk factors.

Our results confirmed several risk factors for PE in the ED setting. Previous studies revealed a strong association of the rising incidence of VTE with obesity^24,25^. Cancer is considered a risk factor for VTE, and about 20% to 30% of first diagnosed VTEs are cancer-associated^26,27^. Previous studies also showed that incidence rates of VTE increase with age^1,7,28,29^. Recognizing these traditional risk factors for PE is important for forming a pre-test probability for PE and facilitating the diagnosis of PE. Our results showed that the Midwest region was associated with a lower proportion of PE ED visits. We hypothesized that this geographic variation might be due to differences in the prevalence of risk factors, racial composition^30^, access to care, and the approach to diagnosing PE.

From 2010 to 2018, the hospitalization rate of PE remained stable, with 66% of patients diagnosed with PE being admitted. Approximately half of the PE patients received heparin treatment (low-molecular-weight heparin included), and only 8.8% received factor Xa inhibitors. A decade ago, Schissler et al. demonstrated a very high admission rate (86%) in patients diagnosed with PE in the US ED^16^. Similarly, less than 10% of patients with a primary (first-listed) diagnosis of PE were treated at home in a study performed using the Nationwide Emergency Department Sample (NEDS) and the National Inpatient Sample (NIS) in the US from 2007 and 2012^31^. A later Canadian study reported about 50% of acute PE patients in the ED were discharged with outpatient treatment^32^. With advances in risk stratification of PE, low-risk PE patients can be identified^33–35^. Patients with acute low-risk PE can be considered as candidates for discharge with outpatient care, which is safe and effective^36–39^. A more recent US study demonstrated that immediate discharge from the ED with prescription of the oral rivaroxaban showed effectiveness and safety in low-risk PE patients^40^. Despite the accumulating evidence on low-risk PE management, the use DOAC remained low in the analysis until 2018. Taken together, our data suggested that outpatient treatment of low-risk PE patients has not been widely adopted in the US ED. Our study highlights the need for ED physicians to stay up-to-date with current guidelines and recommendations for the management of PE. Further research is needed to explore the reasons for the discrepancies between real-world ED management of PE and guideline recommendations.

Regarding the high admission rate, our results showed that both patient and hospital factors were associated with hospitalization. We found that male sex, arrival during the morning shift, and higher triage levels were independently associated with higher admission rates, whereas lower admission rates were associated with the presentation to the ED during fall and winter seasons. Male gender is an independent predictor of 30-day mortality, accounting for 10 points in the PESI scoring system^33^. Furthermore, triage vital signs (e.g., pulse rate, respiratory rate, systolic blood pressure, and oxygen saturation) were independent predictors of 30-day mortality in the PESI or sPESI scoring systems^33,34^. We also found that PE patients who arrived in the ED in the morning were more likely to be admitted. In nonfatal pulmonary embolism, the prevalence in the morning is three times higher than that in the evening^41^. In fatal pulmonary embolism, peak incidence occurs from 5 A.M. to 11 A.M^42^. Due to the increased severity, we speculated that patients with PE occurring in the morning had a higher admission rate. Alternatively, physicians may be more likely to discharge low-risk PE patients in the evening, once inpatient beds were filled. In our study, there were no seasonal differences in the PE visits rates, but the admission rate for PE was lower in the winter and fall. The evidence on the seasonal pattern of PE hospitalization was mixed. A study using data from the US National Hospital Discharge Survey found no seasonal variation in the admission rates of PE^43^, while another study revealed seasonal variation, with decreased PE admissions in cold months^44^. We hypothesized that the decrease in hospitalization of PE in the winter may be due to the concomitantly increased admissions for other cardiovascular diseases. This may deplete the available beds for PE and lead to decreased hospitalizations among ED visits for PE. Taken together, this discrepancy between ED visit rates for PE and the ED admission rates for PE suggested that the reasons for hospital admission were mostly hospital-dependent, such as bed availability. To make ED flow better or decrease ED burden, ED physicians can consider implementing risk stratification tools, such as the PESI or sPESI, to identify low-risk PE patients who may be suitable for outpatient treatment. Immediate discharge from the ED with prescription of oral anticoagulation therapy, such as DOACs, has been shown to be safe and effective in selected low-risk PE patients^40^. Additionally, ED physicians can collaborate with inpatient teams to optimize the use of available hospital beds and ensure timely disposition of patients with PE.

Our study has several strengths. We conducted the study using the NHAMCS data, a nationally representative database that can be generalized broadly. Furthermore, the study period was between 2010 and 2018, making the results more relevant to current clinical practice. However, as with all retrospective studies, this study has several limitations. First, we used the ICD codes for the identification of PE cases, which may be subject to coding errors. Second, although the NHAMCS collected a wealth of information, some clinical granularity was lacking. As such, we were unable to verify whether the PE cases were acute or chronic. We did, however, conduct sensitivity analyses restricting to first-listed PE cases, and the results did not materially change (except for the rising trend in PE visit rates). Third, the exclusion of federal, military, and Veterans Administration hospitals from the NHAMCS may lead to a (small) inclusion bias. Finally, because PE is a relatively rare condition in the ED, our sample size of PE was relatively small. Future research with larger sample size is needed to confirm our findings.

## Conclusions

In summary, in this national study spanning nine years, ED visits for patients with PE continued to rise despite stable CT usage. Given the population aging and accumulating PE cases, the disease burden of PE in the ED may continue its rising trend. Some patients are disproportionately affected by PE, and ED clinicians should be aware of conventional risk factors, which may be key to promptly diagnosing PE in the ED. Hospitalization for PE remains common practice, and certain patient and hospital factors are associated with hospitalization decisions. This study provides important insights into the management of PE in the ED and offers potential strategies for improving patient outcomes and reducing ED burden.

## Supplementary Information

Supplementary Information.

## Data Availability

The datasets used and/or analysed during the current study are available from the corresponding author on reasonable request.

## References

[1] Raskob GE, Angchaisuksiri P, Blanco AN, Buller H, Gallus A, Hunt BJ, Hylek EM, Kakkar A, Konstantinides SV, McCumber M, Ozaki Y, Wendelboe A, Weitz JI (2014). Thrombosis: A major contributor to global disease burden. Arterioscler. Thromb. Vasc. Biol..

[2] Cheuk BL, Cheung GC, Cheng SW (2004). Epidemiology of venous thromboembolism in a Chinese population. Br. J. Surg..

[3] Centers for Disease Control and Prevention (2012). Venous thromboembolism in adult hospitalizations—United States, 2007–2009. Morb. Mortal. Wkly. Rep..

[4] Lee LH, Gallus A, Jindal R, Wang C, Wu CC (2017). Incidence of venous thromboembolism in asian populations: A systematic review. Thromb. Haemost..

[5] Konstantinides SV, Barco S, Lankeit M, Meyer G (2016). Management of pulmonary embolism: An update. J. Am. Coll. Cardiol..

[6] Kempny A, McCabe C, Dimopoulos K, Price LC, Wilde M, Limbrey R, Gatzoulis MA, Wort SJ (2019). Incidence, mortality and bleeding rates associated with pulmonary embolism in England between 1997 and 2015. Int. J. Cardiol..

[7] Ghanima W, Brodin E, Schultze A, Shepherd L, Lambrelli D, Ulvestad M, Ramagopalan S, Halvorsen S (2020). Incidence and prevalence of venous thromboembolism in Norway 2010–2017. Thromb. Res..

[8] Münster AM, Rasmussen TB, Falstie-Jensen AM, Harboe L, Stynes G, Dybro L, Hansen ML, Brandes A, Grove EL, Johnsen SP (2019). A changing landscape: Temporal trends in incidence and characteristics of patients hospitalized with venous thromboembolism 2006–2015. Thromb. Res..

[9] Kasper W, Konstantinides S, Geibel A, Olschewski M, Heinrich F, Grosser KD (1997). Management strategies and determinants of outcome in acute major pulmonary embolism: Results of a multicenter registry. J. Am. Coll. Cardiol..

[10] Stein PD, Matta F, Janjua M, Yaekoub AY, Jaweesh F, Alrifai A (2010). Outcome in stable patients with acute pulmonary embolism who had right ventricular enlargement and/or elevated levels of troponin I. Am. J. Cardiol..

[11] Jones AE, Kline JA (2003). Availability of technology to evaluate for pulmonary embolism in academic emergency departments in the United States. J. Thromb. Haemost..

[12] Huisman MV, Barco S, Cannegieter SC, Le Gal G, Konstantinides SV, Reitsma PH, Rodger M, Vonk Noordegraaf A, Klok FA (2018). Pulmonary embolism. Nat. Rev. Dis. Primers.

[13] Smith SB, Geske JB, Kathuria P, Cuttica M, Schimmel DR, Courtney DM, Waterer GW, Wunderink RG (2016). Analysis of national trends in admissions for pulmonary embolism. Chest.

[14] Lehnert P, Lange T, Møller CH, Olsen PS, Carlsen J (2018). Acute pulmonary embolism in a national danish cohort: Increasing incidence and decreasing mortality. Thromb. Haemost..

[15] Wiener RS, Schwartz LM, Woloshin S (2011). Time trends in pulmonary embolism in the United States: evidence of overdiagnosis. Arch. Intern. Med..

[16] Schissler AJ, Rozenshtein A, Schluger NW, Einstein AJ (2015). National trends in emergency room diagnosis of pulmonary embolism, 2001–2010: A cross-sectional study. Respir. Res..

[17] McCaig L, McLemore T (1994). Plan and operation of the national hospital ambulatory medical survey. Vital Health Stat..

[18] Nawar EW, Niska RW, Xu J (2007). National hospital ambulatory medical care survey: 2005 emergency department summary. Adv. Data.

[19] White RH, Garcia M, Sadeghi B, Tancredi DJ, Zrelak P, Cuny J, Sama P, Gammon H, Schmaltz S, Romano PS (2010). Evaluation of the predictive value of ICD-9-CM coded administrative data for venous thromboembolism in the United States. Thromb. Res..

[20] Schneider D, Appleton L, McLemore T (1979). A reason for visit classification for ambulatory care. Vital Health Stat..

[21] Centers for Disease Control and Prevention Ambulatory Care Drug Database System. https://www2.cdc.gov/drugs/applicationnav1.asp (Accessed 20 September 2021).

[22] DeMonaco NA, Dang Q, Kapoor WN, Ragni MV (2008). Pulmonary embolism incidence is increasing with use of spiral computed tomography. Am. J. Med..

[23] Scheres LJJ, Lijfering WM, Cannegieter SC (2018). Current and future burden of venous thrombosis: Not simply predictable. Res. Pract. Thromb. Haemost..

[24] Kakkar VV, Howe CT, Nicolaides AN, Renney JT, Clarke MB (1970). Deep vein thrombosis of the leg. Is there a "high risk" group?. Am. J. Surg..

[25] Pomp ER, le Cessie S, Rosendaal FR, Doggen CJ (2007). Risk of venous thrombosis: Obesity and its joint effect with oral contraceptive use and prothrombotic mutations. Br. J. Haematol..

[26] Heit JA, Silverstein MD, Mohr DN, Petterson TM, O’Fallon WM, Melton LJ (2000). Risk factors for deep vein thrombosis and pulmonary embolism: A population-based case-control study. Arch. Intern. Med..

[27] Timp JF, Braekkan SK, Versteeg HH, Cannegieter SC (2013). Epidemiology of cancer-associated venous thrombosis. Blood.

[28] Tagalakis V, Patenaude V, Kahn SR, Suissa S (2013). Incidence of and mortality from venous thromboembolism in a real-world population: The Q-VTE study cohort. Am. J. Med..

[29] Alotaibi GS, Wu C, Senthilselvan A, McMurtry MS (2016). Secular trends in incidence and mortality of acute venous thromboembolism: The AB-VTE population-based study. Am. J. Med..

[30] Zakai NA, McClure LA, Judd SE, Safford MM, Folsom AR, Lutsey PL, Cushman M (2014). Racial and regional differences in venous thromboembolism in the United States in 3 cohorts. Circulation.

[31] Stein PD, Matta F, Hughes MJ (2018). National trends in home treatment of acute pulmonary embolism. Clin. Appl. Thromb. Hemost..

[32] Baglin T (2010). Fifty per cent of patients with pulmonary embolism can be treated as outpatients. J. Thromb. Haemost..

[33] Aujesky D, Obrosky DS, Stone RA, Auble TE, Perrier A, Cornuz J, Roy PM, Fine MJ (2005). Derivation and validation of a prognostic model for pulmonary embolism. Am. J. Respir. Crit. Care Med..

[34] Jiménez D, Aujesky D, Moores L, Gómez V, Lobo JL, Uresandi F, Otero R, Monreal M, Muriel A, Yusen RD (2010). Simplification of the pulmonary embolism severity index for prognostication in patients with acute symptomatic pulmonary embolism. Arch. Intern. Med..

[35] Jiménez D, Lobo JL, Barrios D, Prandoni P, Yusen RD (2016). Risk stratification of patients with acute symptomatic pulmonary embolism. Intern. Emerg. Med..

[36] Aujesky D, Roy PM, Verschuren F, Righini M, Osterwalder J, Egloff M, Renaud B, Verhamme P, Stone RA, Legall C, Sanchez O, Pugh NA, N'Gako A, Cornuz J, Hugli O, Beer HJ, Perrier A, Fine MJ, Yealy DM (2011). Outpatient versus inpatient treatment for patients with acute pulmonary embolism: An international, open-label, randomised, non-inferiority trial. Lancet.

[37] Bledsoe JR, Woller SC, Stevens SM, Aston V, Patten R, Allen T, Horne BD, Dong L, Lloyd J, Snow G, Madsen T, Elliott CG (2018). Management of low-risk pulmonary embolism patients without hospitalization: The low-risk pulmonary embolism prospective management study. Chest.

[38] Vinson DR, Mark DG, Chettipally UK, Huang J, Rauchwerger AS, Reed ME, Lin JS, Kene MV, Wang DH, Sax DR, Pleshakov TS, McLachlan ID, Yamin CK, Elms AR, Iskin HR, Vemula R, Yealy DM, Ballard DW (2018). Increasing safe outpatient management of emergency department patients with pulmonary embolism: A controlled pragmatic trial. Ann. Intern. Med..

[39] Yoo HH, Nunes-Nogueira VS, Fortes Villas Boas PJ, Broderick C (2019). Outpatient versus inpatient treatment for acute pulmonary embolism. Cochrane Database Syst. Rev..

[40] Beam DM, Kahler ZP, Kline JA (2015). Immediate discharge and home treatment with rivaroxaban of low-risk venous thromboembolism diagnosed in two US emergency departments: A one-year preplanned analysis. Acad. Emerg. Med..

[41] Sharma GV, Frisbie JH, Tow DE, Yalla SV, Khuri SF (2001). Circadian and circannual rhythm of nonfatal pulmonary embolism. Am. J. Cardiol..

[42] Colantonio D, Casale R, Abruzzo BP, Lorenzetti G, Pasqualetti P (1989). Circadian distribution in fatal pulmonary thromboembolism. Am. J. Cardiol..

[43] Stein PD, Kayali F, Olson RE (2004). Analysis of occurrence of venous thromboembolic disease in the four seasons. Am. J. Cardiol..

[44] Masotti L, Ceccarelli E, Forconi S, Cappelli R (2005). Seasonal variations of pulmonary embolism in hospitalized patients. Respir. Med..

