# Supplementary material for: Pulmonary embolism in United States emergency departments, 2010–2018
Source: Sci Rep. 2023 Jun 5;13:9070. doi: 10.1038/s41598-023-36123-2 (PMC10241783; doi:10.1038/s41598-023-36123-2)
Supplement: Supplementary file 1 — Supplementary Information. [file 41598_2023_36123_MOESM1_ESM.pdf]

Supplemental Table S1. Baseline clinical characteristics of emergency department patients with pulmonary embolism (first-listed), 2010-2018.

**Variable**	**Weighted Number or Weighted Mean**	**Weighted percentage (95% CI)**
Overall		
Age group, n (%)		
18-64	405,000	58.0 (46.7-68.6)
65+	293,000	42.0 (31.4-53.3)
Sex, n (%)		
Male	323,000	46.2 (35.9-56.9)
Female	375,000	53.8 (43.1-64.1)
Race/ethnicity, n (%)		
Non-Hispanic white	484,000	69.3 (58.4-78.4)
Non-Hispanic black	146,000	20.9 (13.2-31.6)
Hispanic	66,000	9.5 (4.6-18.4)
Other	2,000	0.3 (0.0-2.3)
Insurance, n (%)		
Private insurance	223,000	33.7 (24.1-44.8)
Medicare	254,000	38.5 (28.6-49.4)
Medicaid or state-based programs	98,000	14.9 (7.5-27.3)
Self-pay (uninsured)	75,000	11.4 (5.0-24.0)
Other	11,000	1.7 (0.4-7.0)
Season, n (%)		
Spring (Mar. – May)	155,000	22.2 (14.9-31.8)
Summer (Jun. – Aug.)	219,000	31.3 (21.9-42.5)
Fall (Sep. – Nov.)	175,000	25.0 (16.2-36.6)
Winter (Dec. – Feb.)	150,000	21.5 (14.0-31.4)
Weekend, n (%)	243,000	34.8 (25.2-45.7)
Time of ED presentation, n (%)		
7:00 am to 2:59 pm	275,000	40.0 (29.9-51.0)
3:00 pm to 10:59 pm	341,000	49.6 (39.1-60.2)
11:00 pm to 6:59 am	72,000	10.4 (5.5-18.7)
Geographic region, n (%)		
Northeast	226,000	32.4 (22.5-44.3)
Midwest	186,000	26.6 (17.6-38.0)
South	189,000	27.1 (18.3-38.2)
West	97,000	13.9 (8.0-23.0)
Metropolitan area, n (%)	588,000	87.7 (74.6-94.5)
Arrival by ambulance, n (%)	186,000	27.7 (18.6-39.1)
Number of comorbid conditions,	2.0	1.5-2.4

mean (SE)		
Comorbidities, n (%)		
Cancer	53,000	10.0 (5.3-18.1)
Venous thromboembolism	70,000	15.1 (8.0-26.7)
Obesity	90,000	19.4 (11.2-31.6)
Chronic heart failure	51,000	7.3 (4.0-13.2)
Chronic obstructive pulmonary disease	59,000	11.1 (5.9-20.0)
Most common chief complaints, n (%)		
Dyspnea	321,000	46.0 (35.9-56.5)
Chest pain	175,000	25.1 (17.1-35.1)
Triage level, n (%)		
1	13,000	2.3 (0.7-6.8)
2	207,000	37.3 (25.3-51.1)
3	276,000	49.7 (37.2-62.3)
4	60,000	10.8 (4.9-22.0)
5	0	0 (0-0)
Pain score, n (%)		
Severe (7-10)	210,000	41.3 (29.6-54.0)
Moderate (4-6)	83,000	16.4 (9.6-26.5)
Mild (1-3)	20,000	4.0 (1.0-15.1)
No pain (0)	195,000	38.4 (27.7-50.4)
Triage vital signs		
Body temperature, mean, °C	36.8	36.6-36.9
Heart rate, mean, beats per min	96.1	91.7-100.6
Respiratory rate, mean, breaths per min	23.9	17.8-30.0
Oxygen saturation, mean, %	95.4	94.3-96.5
Systolic blood pressure, mean, mmHg	139.4	133.5-145.3
Simplified PESI ≥1[a]	307,000	49.2 (38.4-60.1)
Individual component of sPESI		
Age>80 years	127,000	18.1 (11.0-28.4)
History of cancer	53,000	10.0 (5.3-18.1)
History of chronic cardiopulmonary disease	102,000	14.6 (8.8-23.3)
Heart rate ≥110 beats per min	157,000	23.6 (14.7-35.8)
Systolic blood pressure<100mmHg	37,000	5.6 (2.1-14.1)
Oxygen saturation<90%	82,000	12.1 (5.6-24.4)
ED management, n (%)		

Intubation	0	0 (0)
CPR	0	0 (0)
Chest CT scan[b]	351,000	66.2 (52.6-77.6)
Ultrasound	108,000	15.5 (8.6-26.3)
D-dimer test	240,000	45.2 (32.6-58.4)
Heparin	532,000	76.3 (64.9-84.8)
Coumarins	57,000	8.1 (4.3-15.0)
Factor Xa inhibitors	64,000	9.1 (4.6-17.2)
Length of ED stay, mean, hours	5.8	4.9-6.7
ED disposition, n (%)		
Admission	549,000	78.7 (68.3-86.3)
Died in the ED	4,000	0.5 (0.1-3.7)
Hospitalization[c]		
ICU admission, %	110,000	25.7 (14.6-41.1)
Length of hospital stay, mean, days	5.9	4.1-7.7
Inpatient mortality, n (%)	12,000	2.4 (0.7-7.5)

[a] Available in 123 patients

[b] from 2012-2018

[c] Among those who were hospitalized

Abbreviations: ED = emergency department; sPESI=simplified Pulmonary Embolism Severity Index; CPR = cardiopulmonary resuscitation; CT = computed tomography; ICU = intensive care unit.

Supplemental Table S2. Emergency department visit rates for pulmonary embolism (first-listed), overall, stratified, and multivariable analysis, 2010-2018.

**Variable**	**Proportion of PE, %**	**Adjusted OR (95%CI)***
**Overall**	0.07	
Age group, years		
18-64	0.05	1.0 (reference)
65+	0.15	2.5 (0.96-6.7)
Sex		
Male	0.08	0.9 (0.5-1.6)
Female	0.07	1.0 (reference)
Race/ethnicity		
Non-Hispanic white	0.08	1.0 (reference)
Non-Hispanic black	0.07	0.8 (0.3-1.9)
Hispanic	0.05	0.5 (0.1-2.0)
Insurance		
Private insurance	0.09	1.0 (reference)
Medicare	0.12	0.5 (0.2-1.0)
Medicaid or state-based programs	0.05	0.7 (0.3-1.8)
Self-pay (uninsured)	0.06	1.3 (0.3-5.5)
Other	0.03	0.7 (0.1-3.7)
Season		
Spring (Mar. – May)	0.06	0.6 (0.3-1.2)
Summer (Jun. – Aug.)	0.09	1.0 (reference)
Fall (Sep. – Nov.)	0.07	0.7 (0.3-1.6)
Winter (Dec. – Feb.)	0.07	0.8 (0.3-1.7)
Weekend		
Non-weekend	0.07	1.0 (reference)
Weekend	0.10	**1.9 (1.02-3.7)**
Time of ED presentation		
7:00 am to 2:59 pm	0.07	0.6 (0.3-1.2)
3:00 pm to 10:59 pm	0.09	1.0 (reference)
11:00 pm to 6:59 am	0.05	0.4 (0.1-1.3)
Geographic region		
Northeast	0.14	1.0 (reference)
Midwest	0.08	0.5 (0.2-1.1)
South	0.05	**0.3 (0.1-0.7)**
West	0.05	**0.3 (0.1-0.9)**
Arrival mode		
Arrival not by ambulance	0.07	1.0 (reference)
Arrival by ambulance	0.11	1.4 (0.6-3.1)

Cancer Status		
Cancer	0.07	1.2 (0.5-3.1)
No cancer	0.15	1.0 (reference)
History of VTE		
VTE	0.84	**7.0 (2.9-17.2)**
No VTE	0.07	1.0 (reference)
Obesity status		
Obesity	0.34	**3.9 (2.0-7.6)**
No obesity	0.07	1.0 (reference)

Significant odds ratios are highlighted in bold.

Abbreviations: ED = emergency department; PE = pulmonary embolism; OR = odds ratio; VTE = venous thromboembolism.
*Multivariable model adjusts for all variables in the Table.

Supplemental Table S3. Emergency department admission rates for pulmonary embolism (first-listed), overall, stratified, and multivariable analysis, 2010-2018.

Variable	ED admission rate, %	Adjusted OR (95%CI)*
**Overall**	78.7	
Age group, years		
18-64	78.0	1.0 (reference)
65+	79.5	4.4 (0.3-54.3)
Sex		
Male	85.0	**3.5 (1.1-11.5)**
Female	73.2	1.0 (reference)
Race/ethnicity		
Non-Hispanic white	78.1	1.0 (reference)
Other	79.9	1.3 (0.3-5.7)
Insurance		
Private insurance	69.8	1.0 (reference)
Medicare	79.7	0.3 (0.0-7.8)
Other	88.5	3.0 (0.3-31.9)
Season		
Spring (Mar. – May)	92.3	2.0 (0.2-25.0)
Summer (Jun. – Aug.)	86.6	1.0 (reference)
Fall (Sep. – Nov.)	69.8	0.2 (0.0-1.3)
Winter (Dec. – Feb.)	63.3	**0.1 (0.0-0.9)**
Weekend		
Non-weekend	80.0	1.0 (reference)
Weekend	77.9	0.6 (0.1-3.0)
Time of ED presentation		
7:00 am to 2:59 pm	90.3	13.7 (0.9-211.3)
3:00 pm to 10:59 pm	72.0	1.0 (reference)
11:00 pm to 6:59 am	69.4	5.5 (0.5-66.0)
Geographic region		
Northeast	83.5	1.0 (reference)
Midwest	77.7	3.2 (0.4-24.7)
South	81.7	16.2 (0.6-430.6)
West	63.2	2.0 (0.3-13.7)
Arrival mode		
Arrival not by ambulance	77.3	1.0 (reference)
Arrival by ambulance	80.0	1.0 (0.2-4.7)
Triage level		
1 & 2	92.1	**18.7 (2.4-145.0)**
3	77.3	1.0 (reference)

4 & 5	60.5	0.9 (0.2-5.2)

Significant odds ratios are highlighted in bold.

Abbreviations: ED = emergency department; OR = odds ratio.
*Multivariable model adjusts for all variables in the Table.

Supplemental Fig. S1.

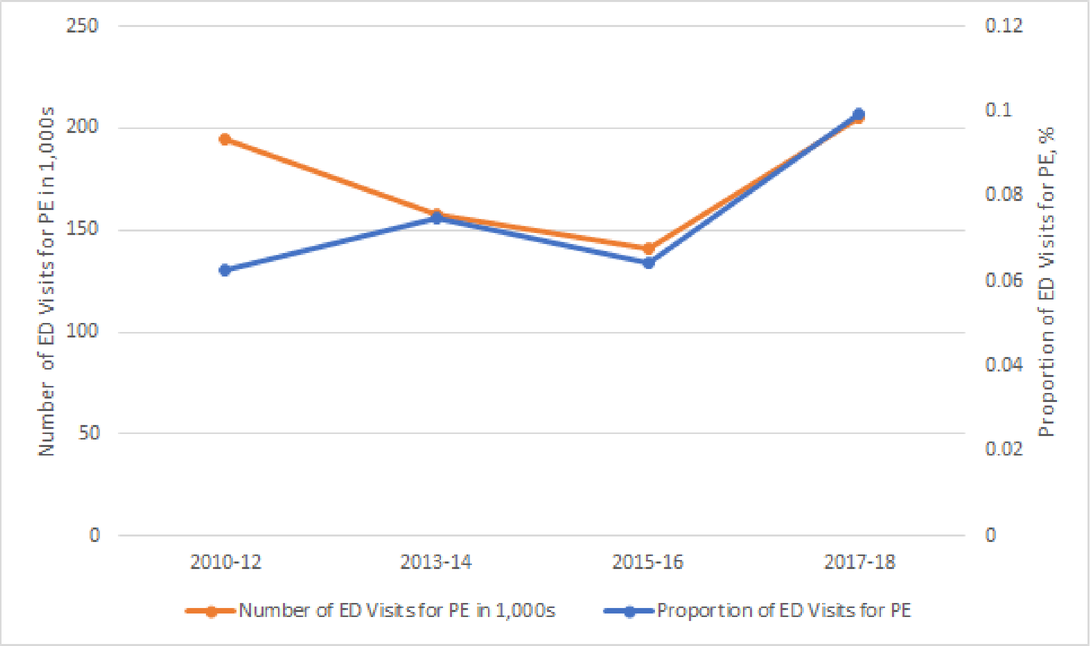

Supplemental Fig. S2.

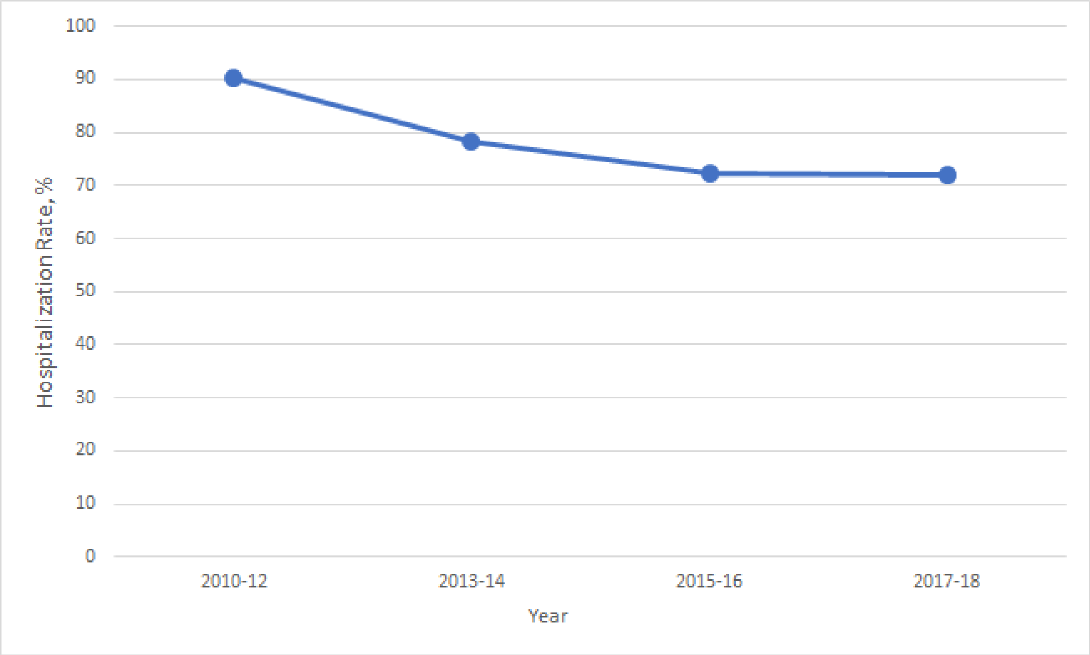

Supplemental Fig. S3.

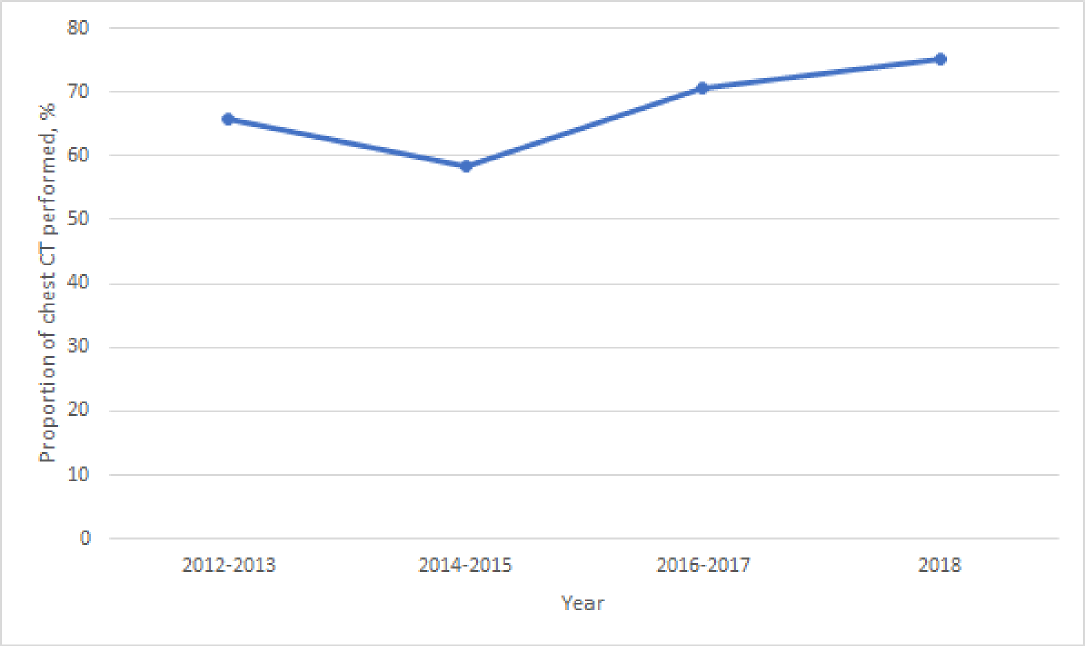

FIGURE LEGENDS

Supplemental Fig. S1. The number and proportion of emergency department visits for pulmonary embolism (first-listed), 2010-2018.

Supplemental Fig. S2. The hospitalization rate among emergency department visits for pulmonary embolism (first-listed), 2010-2018.

Supplemental Fig. S3. The proportion of chest computed tomography scans performed among emergency department visits for pulmonary embolism (first-listed), 2010-2018.
